# Enhancing customer satisfaction with chatbots: The influence of communication styles and consumer attachment anxiety

**DOI:** 10.3389/fpsyg.2022.902782

**Published:** 2022-07-22

**Authors:** Ying Xu, Jianyu Zhang, Guangkuan Deng

**Affiliations:** ^1^School of Economics and Management, Southwest University of Science and Technology, Mianyang, China; ^2^School of Business Administration, Southwestern University of Finance and Economics, Chengdu, China

**Keywords:** chatbot, communication styles, consumer attachment anxiety, warmth perception, customer satisfaction

## Abstract

Chatbots are increasingly occupying the online retailing landscape, and the volume of consumer-chatbot service interactions is exploding. Even so, it still remains unclear how chatbots should communicate with consumers to ensure positive customer service experiences and, in particular, to improve their satisfaction. A fundamental decision in this regard is the choice of a communication style, specifically, whether a social-oriented or a task-oriented communication style should be best used for chatbots. In this paper, we investigate how using a social-oriented versus task-oriented communication style can improve customer satisfaction. Two experimental studies reveal that using a social-oriented communication style boosts customer satisfaction. Warmth perception of the chatbot mediates this effect, while consumer attachment anxiety moderates these effects. Our results indicate that social-oriented communication style can be beneficial in enhancing service satisfaction for highly anxiously attached customers, but it does not work for the lowly anxiously attached. This study provides theoretical and practical implications about how to implement chatbots in service encounters.

## Introduction

Brands are increasingly using chatbots to supplement and even replace human agents in service interactions (Roy and Naidoo, 2021). It is estimated that as many as one-third of online interactions involve a chatbot and this proportion is expected to increase particularly in a (post-) COVID environment (Hollebeek et al., 2021; Shumanov and Johnson, 2021). Facilitated by recent advancement in artificial intelligence (AI) and natural language processing, these agents can deliver services similar to human agents, in addition to offering multiple benefits, such as convenience, 24/7 availability, immediate responses (Thomaz et al., 2020; Gelbrich et al., 2021), and cost reduction for brands (Sands et al., 2020). Despite the prevalence of chatbots in business practices, consumers still remain sceptical and reluctant to engage with them (Van Pinxteren et al., 2020), as shown by research reporting a higher preference for human interaction, as compared to chatbot-based conversations (Adam et al., 2021). Nonetheless, commercial interest in chatbot technology remains high, due to their aforementioned benefits (Thomaz et al., 2020). Thus, addressing consumer scepticism is of critical importance, which forces brand managers to consider how chatbots should be designed to ensure positive customer service experiences (Sands et al., 2020; Roy and Naidoo, 2021).

However, the related research is limited due to the nascency of chatbot technology (Roy and Naidoo, 2021). The few studies to date suggest that brand manager should enhance the humanness of chatbots, and has examined how identity cues (e.g., human name), visual cues (e.g., human figure), genders, and conversational cues (e.g., conversation skill) shape consumer attitudes and behaviors (Araujo, 2018; Go and Sundar, 2019; Schuetzler et al., 2020; Sheehan et al., 2020; Borau et al., 2021; Hildebrand and Bergner, 2021; Shumanov and Johnson, 2021). However, despite calls for further work to unpack how to calibrate the communication style used by chatbots to optimize customer experience (Bleier et al., 2019), a simple and more fundamental feature of consumer-chatbot service interactions has been relatively unexplored: the chatbot’s communication style.

In this research, we address these research gaps by focusing on how a chatbot’s communication style affects customers’ service experiences. Specifically, we argue that a chatbot’s social-oriented communication style boosts customer satisfaction and customers’ warmth perceptions of the chatbot mediate this effect. We further argue that the warmth perceptions of the chatbots are contingent on an individual’s attachment anxiety.

Our findings make three main contributions. First, we contribute to the customer service literature by extending the investigation on communication style effect to chatbot service interactions and revealing the psychological process driving the impacts and, more generally, to the hot topic regarding how consumers react to AI that is used to establish and maintain a relationship (Huang and Rust, 2021). Second, we further add to the growing chatbot humanness perception literature and answer calls for investigating more anthropomorphic design cues to enhance chatbot humanness (Go and Sundar, 2019; Schuetzler et al., 2020; Adam et al., 2021). Finally, the current research contributes to the attachment literature by extending the investigation on attachment anxiety effects to chatbot service interactions and demonstrating the attachment patterns developed early in life can guide consumer preferences for chatbots’ specific communication style.

## Literature review

### Chatbots

Chatbots are natural language computer programs that simulate human language and interact with customers with the aid of a text-based dialog (Zumstein and Hundertmark, 2017). In contrast to service robots that have embodiments, they have no embodiment and are only visible to consumers through text in a live chat, resembling SMS exchanges (Söderlund and Oikarinen, 2021). In addition, as they can mimic interpersonal conversations, they are capable of engaging customers on a social level, which distinguishes them from self-service technologies (van Doorn et al., 2017; Pizzi et al., 2021). Particularly, today’s chatbots providing customer service are low-end feeling AI applications, can learn and adapt only to a minimal degree, and do some relationalization, but in a rather mechanical way (Huang and Rust, 2021).

Scholars and designers have aimed at enhancing the humanness of chatbots for a long time (Schuetzler et al., 2020; Roy and Naidoo, 2021), and have found that adding human attributes to chatbots can enhance positive experiences, and trigger social and emotional connectedness (Araujo, 2018; Adam et al., 2021). Prior research also examines how visual (human figure) and identity cues (human name or identity) shape customer attitudes and behaviors (Araujo, 2018; Van den Broeck et al., 2019), arguing that identity cues have primacy over other humanness cues, such as language. As shown in Table 1, scholars recently began paying attention to the design of discourse and communication styles to enhance chatbot humanness (Go and Sundar, 2019; Roy and Naidoo, 2021). They suggest that human-like language, message interactivity, conversation skills, emotional support, and conversational styles are all useful (Go and Sundar, 2019; Schuetzler et al., 2020; Sheehan et al., 2020; Gelbrich et al., 2021; Roy and Naidoo, 2021; Shumanov and Johnson, 2021).

**Table 1:** Comparison of related literature.

Study	Main independent variables	Dependent variable	Contribution	Domain	Consumer individual differences
Araujo (2018)	– Language style and name – Framing	– Anthropomorphism – Social presence – Attitude – Satisfaction – Emotional connection	Human-like cues, such as human-like language and name, influence anthropomorphism and company-related outcomes, including attitudes toward, as well as satisfaction and emotional connection with the company.	Computer Science	No
Bleier et al. (2019)	– Linguistic style	– Social presence – Entertainment – Informativeness – Sensory appeal	Using a conversational (vs. journalistic) linguistic style drives entertainment and social presence.	Marketing	No
Go and Sundar (2019)	– Anthropomorphic visual cues – Message interactivity – Identity cues	– Attitude – Chatbot evaluation – Behavioral intention	Message interactivity influences users’ chatbot evaluation, attitudes towards the website, and behavioral intentions. Anthropomorphic visual cues compensate for the impersonal nature of chatbots that have low message interactivity. Identity cues set expectations for chatbot performance.	Computer Science	No
Hildebrand and Bergner (2021)	– Turn-taking – Social cues	– Affective trust – Firm perception – Investor behavior	Turn-taking and social cues enhance affective trust in a robo advisor, firm perception, and recommendation acceptance.	Financial marketing	No
Schuetzler et al. (2020)	– Conversation skills: Tailored response and response variety	– Humanness perception	Conversation skills enhance humanness perceptions of chatbots	Information System	No
Sheehan et al. (2020)	– Seeking clarification	– Adoption intent	Seeking clarification has a positive effect on adoption intent.	Marketing	Need for human interaction
Shumanov and Johnson (2021)	– Personality	– User engagement – Purchasing behavior	A congruent consumer-chatbot character has positive impacts on user engagement and purchasing behavior	Computer Science	No
Gelbrich et al. (2021)	– Emotional support	– Customer satisfaction -Persistence	Emotional support positively affects customer satisfaction and persistence to use the agent.	Marketing	No
Roy and Naidoo (2021)	– Conversational Style: Warm versus competent	– Attitudes toward brand – Purchase intention	Present-oriented customers prefer a warm versus competent chatbot conversation, while future-oriented customers prefer a competent conversation, leading to favorable brand attitudes and purchase intentions.	Marketing	Time orientation
Current research	– Communication style: social-oriented vs. task-oriented	– Customer satisfaction	Using a social-oriented communication style increases customer satisfaction, an effect that warmth perception mediates this effect, while the consumer attachment anxiety moderates this effect.	Marketing	Attachment anxiety

Importantly, communication style is the most controllable factor for the development of chatbots (Thomas et al., 2018; Thomaz et al., 2020). There are many potentially relevant dimensions along which communication styles vary that can influence consumers’ responses. Related research has examined specific dimensions. Bleier et al. (2019) examine web design and demonstrate that chatbot’s conversational tone (vs. journalistic tone) is a key driver of social presence. Roy and Naidoo (2021) engage warmth which emphasizes traits like friendliness and helpfulness and competence to the conversational style of chatbots, and unveil how they affect consumer attitudes toward the brand and purchase intentions. However, as mentioned above, many chatbots attempt to establish a relationship with customers, characterized by informal and relational dialog with social interactions such as customary greetings, emotional concerns, social praise, and well-wishing (Wilson-Nash et al., 2020), which is particularly relevant with social-orientation and prior studies do not capture. Also, we still know very little about how consumers react to AI that is used to form and maintain relationships (Huang and Rust, 2021). Therefore, we focus on two communication style dimensions, task-oriented and social-oriented, to frame a chatbot’s communication style.

### Chatbot’s communication style

Communication style is an important topic in business, due to its close relevance to sales (Williams and Spiro, 1985), patronage intention (Keeling et al., 2010), brand trust (Gretry et al., 2017), and customer satisfaction (Van Dolen et al., 2007). Prior work has investigated it in various human-human interactions, such as sales interaction, online group interaction, and social media interaction. To extend this large body of literature, we examine chatbot’s communication style and its impact on consumer responses. Specifically, a chatbot with a social-oriented interaction style is more personal, focuses on satisfying consumers’ emotional needs and establishing relationships when offering help, and is often characterized by informal and relational dialog with social interactions such as customary greetings, small talk, emotional concerns, social praise, and well-wishing. By contrast, task-oriented communication style is highly goal-oriented and purposeful, focuses on task efficiency, works hard to complete task, and the conversation is more formal and involving purely on task dialog (Williams and Spiro, 1985; Van Dolen et al., 2007; Keeling et al., 2010; Chattaraman et al., 2019). In particular, these social interactions can enhance the closeness, beyond just signaling warmth. From a customer’s perspective, both communication styles may satisfy the customer’s utilitarian needs by providing product-related information and answering questions, while a social communication style may also meet certain social needs of the customer but take up unnecessary time.

Related research has examined the communication styles of avatars, which refer to visual representations of an entity (Holzwarth et al., 2006). Keeling et al. (2010) examine sales avatars and find that social- and task-oriented communication styles both contribute to consumer trust and patronage intentions. Foster et al. (2021) examine brand avatar and show that social interactions by the brand avatar facilitate the consumer-brand relationship. Together, these findings shed light on the important role of communication styles in shaping customers’ responses, but it still remains unclear which specific communication style is more favorable.

People treat the chatbot as a social actor and mindlessly apply social rules to respond to them when chatbots possess social cues such as conversation and interactivity (Nass and Moon, 2000; Kim and Sundar, 2012). Therefore, when customers interact with chatbot through a live chat, the two fundamental dimensions of social cognition theory—warmth and competence—offer a theoretical framework for examining the role of chatbot communication style in consumer responses. Next, we provide a brief overview of these two dimensions.

### Dimensions of social cognition

The social cognition literature has identified warmth and competence as the two fundamental dimensions (Fiske et al., 2007), and prescribes that people rely primarily on these two dimensions to infer others’ intent and ability. The warmth dimension captures perceived friendliness, helpfulness, and trustworthiness, while the competence captures perceived intelligence, skillfulness, and capability (Cuddy et al., 2008).

Previous service research has shown the fundamental mechanisms of social cognition, in particular warmth and competence, in interpersonal service interaction (e.g., Scott et al., 2013; Li et al., 2019). Importantly, van Doorn et al. (2017) propose that these two dimensions can mediate the relationship between automated social presence (ASP) and several key service and customer outcomes (i.e., satisfaction, loyalty, engagement, and wellbeing), and ASP refers to the extent to which technologies (e.g., robots) create feelings of social presence. As chatbot can imitate interpersonal interactions and engage consumers on a social level, thus, the two fundamental dimensions should be relevant to understand consumers’ responses to chatbot communication style.

Although both dimensions should be necessary for social cognition, previous research has focused on the primacy of one dimension over the other (e.g., primacy of warmth dimension, Abele and Wojciszke, 2014; primacy of competence dimension, Aaker et al., 2010). In a consumer-chatbot service interaction, the homogeneous nature of a chatbot’s service delivery can lead to the lack of social and emotional value (Sands et al., 2020), this might induce consumers to pay more attention and attach more weight to warmth (vs. competence) dimension. However, several factors can moderate the primacy of warmth. For example, warmth (vs. competence) can be more critical when communal relationship norm is salient (Li et al., 2019), or when consumers focus on having a satisfying consumption experience (Wang et al., 2017), or when consumers hold a future orientation (Roy and Naidoo, 2021). To extend this stream of research, this study considers consumer attachment anxiety as a contingency variable, given its better prediction for customers’ preference for closeness (warmth; Mende et al., 2013).

### Attachment anxiety

Attachment anxiety is a fundamental dimension of attachment styles viewed as the systematic pattern of relational expectations and social behaviors (Mikulincer and Shaver, 2016), the other is attachment avoidance. Attachment anxiety refers to the extent to which a person fears interpersonal rejection or abandonment, has an excessive need for approval from others, and distress when a relationship partner is unavailable in times of need. Recent work suggests that attachment anxiety might be more relevant to understand how consumers react to closeness. For example, Kogut and Kogut (2011) show that attachment anxiety encourages the endowment effect, while attachment avoidance has no significant effect. Similarly, Keefer et al. (2012) find that attachment anxiety rather than attachment avoidance predicted attachment to objects. Konok et al. (2016) show that attachment anxiety, but not attachment avoidance, associates with attachment to mobile device. Gasiorowska et al. (2022) find that individuals with high (vs. low) displayed a higher propensity to purchase status-signaling goods as a substitute for attachment, while the relationship between attachment avoidance and status consumption was unclear. Particularly, Pozharliev et al.’s (2021) study regarding service robot suggests that attachment anxiety is more relevant. Moreover, given that a typical response to anxiety is investment in alternative, non-human sources, while a typical response to attachment avoidance is resisting emotional dependence on other people (Keefer et al., 2012), attachment anxiety is more relevant to the current study. Therefore, we do not have particular expectations regarding the interplay of attachment avoidance with chatbot’s communication styles.

## Hypothesis

### The main effect of social-oriented communication style

We reason that the use of a social-oriented communication style can exert positive effects on customer satisfaction. In person-to-person service delivery, a service’s personalization is a major concern, due to its centrality in a consumer’s ultimate satisfaction with a particular service (Surprenant and Solomon, 1987). Given that engaging human attributes for chatbots encourages consumers to perceive them as humans and hold expectations that resemble those they hold for people (Araujo, 2018; Gelbrich et al., 2021), consumers desire to have a personalized service when engaging with service chatbots. Particularly, as the homogeneous nature of a chatbot’s service delivery can lose emotional and social value, personalizing this service delivery becomes more important for the customer service experience. To achieve this, personalizing the conversation through communication style is an option (Thomaz et al., 2020; Wilson-Nash et al., 2020). Specifically, compared to a task-oriented communication style, a social-oriented communication style is more personal and makes each customer feel like an individual, not just another customer (Van Dolen et al., 2007). This coincides with Surprenant and Solomon’s (1987) programmed personalization which focuses on personalizing the interactive process of a service by encouraging small talk, using customary greetings, etc. Furthermore, these authors demonstrate that programmed personalization exerts strong positive effects on customer satisfaction. In addition, experiential communication characterized by the integration of sensory, emotional, and social information, can create affective customer-brand connections (Moffett et al., 2021). Thus, compared to using a task-oriented communication style, a social-oriented communication style, expressing emotional and social concerns, is more experiential and engaging, and can enhance customer satisfaction. As such, we hypothesize the following:

*H1*: Customers indicate a higher level of customer satisfaction when chatbots employ a social-oriented communication style than a task-oriented communication style.

### The mediating effect of warmth perception

Next, we propose that a social-oriented communication style may enhance customer satisfaction through warmth perception. People rely on these dimensions of social cognition to infer perceptions of other people and non-human entity, such as products, brand, money, and service robots (Fiske et al., 2007; Aaker et al., 2010; Zhou et al., 2018; Choi et al., 2020). When engaging human attributes for chatbots in consumer-chatbot interactions, consumers tend to treat them and unconsciously apply social rules in their responses (Kim and Sundar, 2012). As a result, the two fundamental dimensions of social cognition become more relevant in this service interaction if the chatbot possesses social cues such as conversation, interactivity, and social roles. For example, Araujo (2018) shows that chatbot’s humanlike name makes it more likeable and personal. Similarly, Hildebrand and Bergner (2021) find that chatbot’s turn-taking during the initial onboarding phase can lead to greater levels of affective trust in the chatbot. Thus, when a chatbot interacts with customers through a live chat in the format of Q&A, the two fundamental dimensions of social perception can be employed as a theoretical framework for examining consumers’ responses to different communication styles.

We propose that using a social-oriented communication style increases perceptions of warmth, because the latter is associated with a person’s emotional and interpersonal characteristics (Yang et al., 2020), and this communication style communicates emotional concern and is relationship orientation (Williams and Spiro, 1985). Moreover, prior studies reveal that individuals use informal conversation with familiar people, such as friends and family (Gretry et al., 2017); consequently, a social-oriented communication style, characterized by the use of informal conversation, can cognitively ignite the perception of warmth related to friends and family. However, consumers may have a similar competence perception for a chatbot using social-oriented or task-oriented communication styles, because they both convey the same amount of information (Bleier et al., 2019). As a result, perceptions of competence will not differ between the two communication styles. In fact, prior research suggests that warmth has a more decisive influence on affective responses than competence (Fiske et al., 2007; Gelbrich et al., 2021).

Warmth perception, in turn, can improve customer satisfaction. On the one hand, warmth perception relies on the inference of good intentions (Gelbrich et al., 2021). The higher the good intention evaluation, the more satisfied the customer will feel with regards to not being cheated. On the other hand, warmth perception is associated with considering others’ needs (Yang et al., 2020). Thus, the higher warmth perception, the more customers will feel cared for and understood, which contributes to customer satisfaction (Packard et al., 2021). Additionally, warmth perception can compensate for the lack of actual warmth in a chatbot’s service delivery, which is homogeneous in nature and often perceived as cold and impersonal (Sands et al., 2020). Furthermore, the marketing and service literature also reveals that warmth perception is associated with customer satisfaction (van Doorn et al., 2017; Li et al., 2019). For example, in a study on emoticon use in online service encounters, Li et al. (2019) show that consumers perceived service employees who use emoticons as warmer and are therefore more satisfied. Similarly, Choi et al. (2020) reveal that people perceive humanoid service robots as warmer than non-humanoids and are thus more satisfied.

In sum, we reason that consumers interacting with a chatbot that employs a social-oriented communication style will feel warmer, and therefore be more satisfied. Thus, we hypothesize that:

*H2*: Warmth perceptions of the chatbot mediate the relationship between chatbots’ social-oriented communication style (vs. a task-oriented communication style) use and customer satisfaction.

### The moderating effect of attachment anxiety

We propose that consumer attachment anxiety moderates the indirect effect of a social-oriented communication style on satisfaction *via* warmth perception. Specifically, we argue that the moderating effect occurs because of the more accessible and diagnostic warmth perception among customers with high attachment anxiety. High anxiety customers expect more social care, they thus expect a chatbot as their social partner to provide more emotional concerns and to be more responsive and intimated. Because the warmth dimension associates with one’s kindness and good-naturedness, which involves the consideration of others’ needs, customers in this condition should put more emphasis on cues eliciting warmth perceptions, leading them to evaluate the chatbot dominantly based on warmth perception. In particular, when a chatbot uses a social-oriented communication style, which is more congruent with these customers’ expected behaviors, customers’ warmth perception of the chatbot could be dramatically improved since closeness underlies this communication style. However, for low anxiety customers, the warmth perception is less accessible and diagnostic; consequently, we expect that customers’ warmth perception of the chatbot might not differ between social-oriented and task-oriented communication style use. Thus, we predict that:

*H3*: Attachment anxiety moderates the effect of chatbots’ social-oriented communication style (vs. task-oriented communication style) use on customer satisfaction through the mediation of warmth perception of chatbots. Specifically, this effect is stronger for high anxiety consumers compared to its low counterpart.

**Figure 1:**
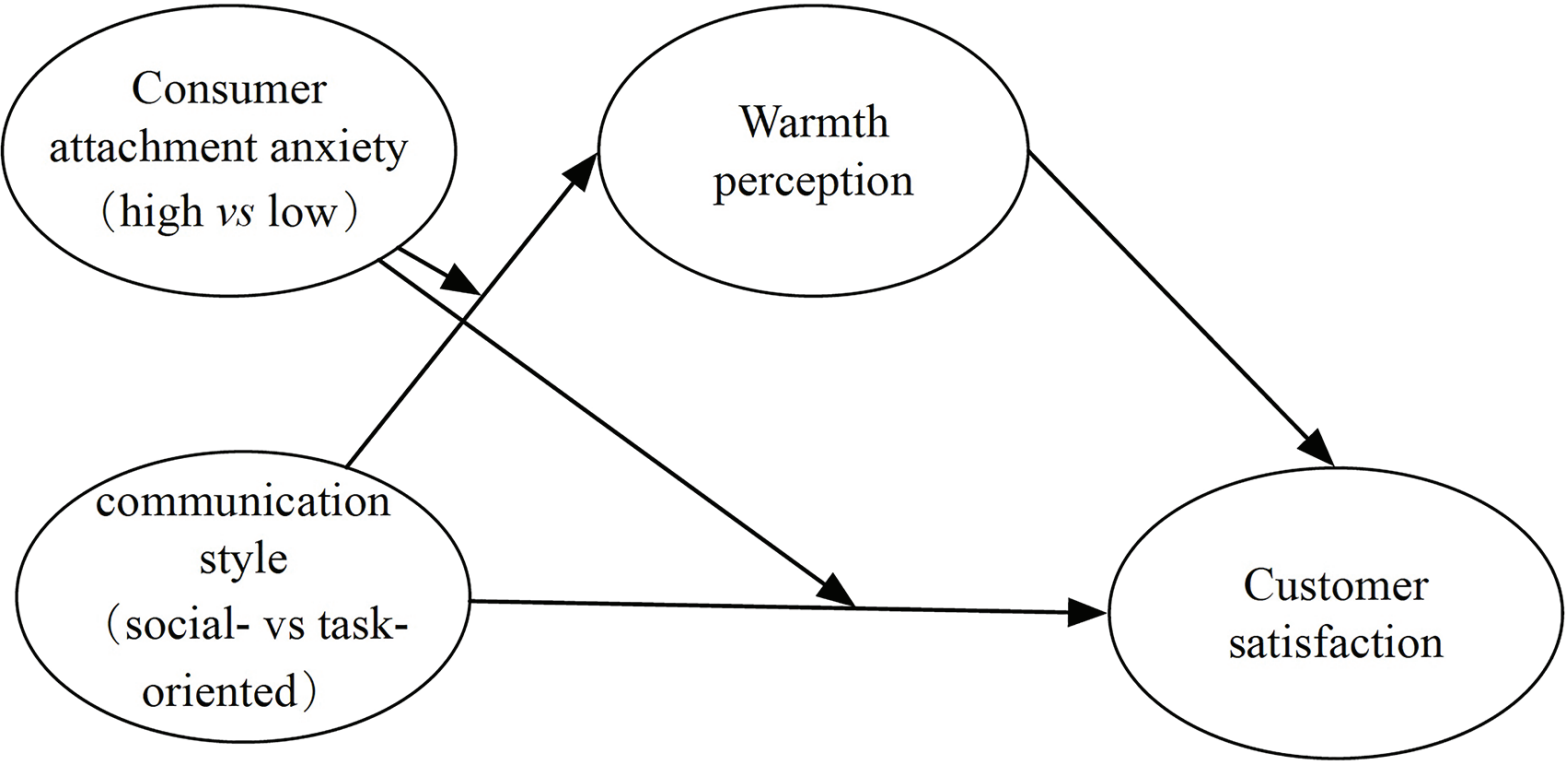
Conceptual model.

To conclude this section, Figure 1 summarizes our conceptual framework.

## Methods and results

### Study 1

Study 1 is a scenario-based experiment aimed to test H1 and H2 where respondents were presented with a conversation screenshot with a chatbot which using a social-oriented or a task-oriented communication style and were asked to imagine themselves as the customer. The study also seeks to rule out competence perception as a theoretical mediator. Based on prior work, we controlled for age and gender of participants (Sheehan et al., 2020; Gelbrich et al., 2021; Roy and Naidoo, 2021), as these factors may bias our results.

#### Method

##### Participants

A total of 142 undergraduate students from a large university in Mianyang, China, participated in this study in exchange for a nominal payment, of which 64.1% were female, ranging in age from 19 to 24 years (see Appendix B for sample characteristics). Informed consent was obtained from all individual participants included in the study. No identifiable information was recorded. This sample size has a power greater than 0.8 to detect medium effect sizes equivalent to ƒ = 0.25, assuming a conventional alpha level of 0.05 (Cohen, 1988).

##### Study design and procedure

The experimental design was a one-factor (social-oriented vs. task-oriented communication style) between-subject design. We first developed two different scenarios of chatbots engaging in task- vs. social-oriented communication styles around a fictitious sports brand: M. Scenario-based manipulation has been engaged by prior work (Go and Sundar, 2019; Roy and Naidoo, 2021). Moreover, prior work has engaged screenshots of conversations between a customer and chatbot, rather than a live chat (Cohen, 2018; Sheehan et al., 2020). Based on this literature, this study, thus, engages conversation screenshots (Cohen, 2018; Sheehan et al., 2020; Roy and Naidoo, 2021) for task- and social-oriented communication styles manipulation. Based on the literature, the social-oriented communication scenario is characterized by informal and social conversation, including customary greetings, emotional concerns, social praise, and well-wishing, whereas, the task-oriented communication scenario uses formal conversation, aims at completing the task, and no social conversation except the initial greeting. We also ensured that our communication style manipulation was consistent with existing chatbot communication practices in online retailing by using expressions from real customer-chatbot conversations. The scenarios were presented in Chinese, and the English versions of each scenario are presented in Appendix A. Importantly, in contrast to prior research, we used the brand logo (i.e., M) instead of a human figure for the chatbot to avoid the confounding effects of human identity.

Following an extant procedure (Roy and Naidoo, 2021), we engaged the participants in an exercise that involved shopping for a pair of sports shoes. While they pretended to shop on the M sports website, two different communication styles of chatbot conversations involving the purchase of a pair of sports shoes were randomly assigned and presented. They were asked to read the assigned scenario as if they were the customers in the scenario. After they read the assigned scenario, participants were asked to complete a questionnaire that included measures of customer satisfaction, warmth perception, and demographics.

##### Measurements

Adapted from Evans et al. (2000), we assessed satisfaction with a three-item, seven-point semantic different scale, ranging from strongly disagree to strongly agree (*α* = 0.94). Adapted from Cuddy et al. (2008), we measured chatbot warmth perception on a four-item seven-point semantic differential scale (*α* = 0.97). Basing ourselves again on Cuddy et al. (2008), we measured chatbot competence perception on a seven-point semantic differential scale (*α* = 0.88). All measures used in this study are presented in full in Appendix E.

##### Stimuli validation

A stimuli validation pretest was conducted to assess the communication styles of the chatbot. Independent sample population of 40 undergraduate business students from a large university in Mianyang, China (67.5% females; M_age_ = 20), were randomly assigned to one of the two conditions and were asked to evaluate the chatbot’s social- and task-orientation, responding on an eight-item, seven-point scale (1 = strongly disagree, 7 = strongly agree), adapted from Williams and Spiro (1985), as well as Van Dolen et al.’s (2007) measurements for social- and task-oriented communication styles, and we also included a realism check measure. This sample size has a power greater than 0.8 to detect large effect sizes equivalent to ƒ = 0.65, assuming a conventional alpha level of 0.05 (Cohen, 1988).

As expected, the respondents rated social-orientation higher in the social-orientated condition [M_social_ = 5.66, SD_social_ = 0.55; M_task_ = 3.46, SD_task_ = 0.92; *t*(38) = −9.208, *p* < 0.001, Cohen’s *d* = 2.91], and task-orientation higher in the task-oriented condition [M_social_ = 4.13, SD_social_ = 0.99; M_task_ = 5.26, SD_task_ = 0.89; *t*(38) = 3.824, *p* < 0.001, Cohen’s *d* = 1.21], and the respondents perceived the scenarios as highly realistic across the communication style manipulations [M_social_ = 5.97, SD_social_ = 0.67; M_task_ = 5.58, SD_task_ = 0.62; *t*(38) = −1.884, *p* = 0.067, Cohen’s *d* = 0.596]. In addition, independent *t*-test showed that gender had no relationships with the manipulation check (*p* > 0.1).

#### Results

##### Correlation analysis

As Table 2 shows, customer satisfaction was significantly positive correlated with warmth perception (*r* = 0.74, *p* < 0.01) and competence perception (*r* = 0.28, *p* < 0.01), warmth perception was significantly correlated with competence perception (*r* = 0.21, *p* < 0.05).

**Table 2:** Descriptive statistics and correlations for Study 1.

Variables	Mean	Standard deviation	Minimum	Maximum	1	2	3
1. Warmth perception	4.88	0.79	2.25	6.00	1.00		
2. Competence perception	5.56	0.71	4.50	6.00	0.21^*^	1.00	
3. Customer satisfaction	5.14	0.68	3.33	6.00	0.74^**^	0.28^**^	1.00

^*^p < 0.05, ^**^p < 0.01.

##### Customer satisfaction

**Figure 2:**
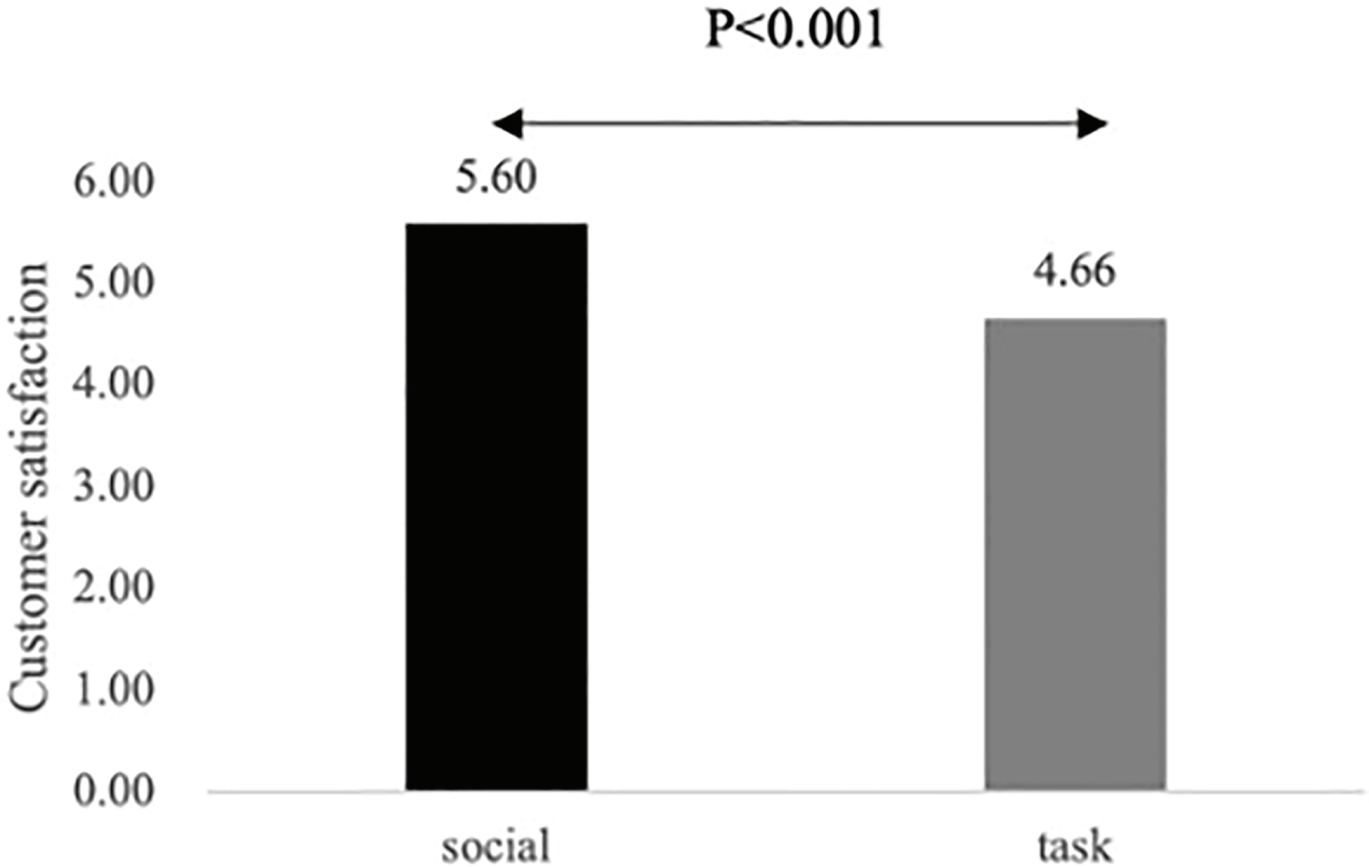
Mean values for customer satisfaction for Study 1.

An ANOVA with customer satisfaction as the dependent variable, the communication style manipulation as the independent variable, and the two controls, revealed a significant main effect of communication style [M_social_ = 5.60, SD_social_ = 0.53; M_task_ = 4.66, SD_task_ = 0.45; *F*(1, 138) = 129.45, *ŋ_p_*^2^ = 0.48, *p* < 0.001], supporting H1 (see Figure 2). The control variables are non-significant (*p* > 0.05).

##### Mediation analysis

To test our mediation hypothesis H2 and rule out competence as a theoretical mediator, we engaged a parallel multiple mediator model (PROCESS model 4; Hayes, 2018) with both warmth and competence perceptions as mediators for the effect of chatbots’ communication style on customer satisfaction. The results confirm that warmth perception mediates the social-oriented communication style’s effect on satisfaction [indirect effect = 0.39, SE = 0.12, 95% CI (0.17, 0.59)]. Social-oriented communication style makes the chatbot appear warmer (*β* = 1.02, SE = 0.10, *t* = 10.01, *p* < 0.001), which boosts satisfaction (*β* = 0.38, SE = 0.06, *t* = 5.92, *p* < 0.001), supporting H2. However, competence perception did not mediate the effect of the social-oriented communication style on satisfaction [indirect effect = 0.008, SE = 0.0088, 95% CI (−0.014, 0.024)].

Study 1 provides direct causal evidence that using a social-oriented communication style increases customer satisfaction, as the participants felt more satisfied when the chatbot used this form of communication style. Further, Study 1 offers initial evidence pertaining to the underlying processes within this effect. Specifically, social-oriented communication increases customer satisfaction, because customers feel more warmth from the chatbot. Finally, Study 1 casts doubt on an alternative explanations, showing that competence perceptions can be ruled out as a concurrent theoretical mediator. We do not propose that this process does not play a role, but, as Moffett et al. (2021) suggest, customers may be less concerned with competence in the early stages of a customer-brand relationship and we only investigate a snapshot of this stage of a relationship.

### Study 2

Study 2 aims to test the potential moderating effect of attachment anxiety as proposed in H3. Moreover, to enhance the experiment’s external validity, this study chooses a different setting, includes two additional control variables, and involves non-student samples. In summary, Study 2 further assesses the consistency of our predicted phenomenon and extends it by investigating the moderating effect of attachment anxiety. We control for the same variables as Study 1 (i.e., age, gender).

#### Method

##### Participants

A total of 185 respondents were recruited from Wenjuanxing, a platform providing functions equivalent to Amazon Mechanical Turk participate in this study in exchange for a nominal payment, of which 52% were female and 48% were non-student (see Appendix C for sample characteristics). Informed consent was obtained from all individual participants included in the study. No identifiable information was recorded. This sample size has a power greater than 0.9 to detect medium effect sizes equivalent to ƒ = 0.25, assuming a conventional alpha level of 0.05 (Cohen, 1988).

##### Study design and procedure

Once again, the experimental design was a one-factor (social-oriented vs. task-oriented communication style) between-subject design. We followed the same communication styles manipulation and chatbot identity as Study 1 (brand logo, rather than human figure). Similar to Study 1, the respondents were asked to imagine shopping online for a pair of jeans from a fictitious brand (MK), but this time we named the chatbot Joe. Importantly, the brand logo is letters “MK.” After exposing to the scenario, participants answered questions on customer satisfaction, warmth perception, consumer attachment anxiety, consumer attachment avoidance, manipulation checks, scenario realism, prior experience, and demographics.

##### Measurements

We used the same customer satisfaction (*α* = 0.87), warmth perception (*α* = 0.94), and communication styles (*α* = 0.87) measures as in Study 1. We used Wang et al.’s (2017) scale to measure consumer attachment anxiety (*α* = 0.94) and consumer attachment avoidance (*α* = 0.86). All measures used in this study are rated on a seven-point semantic differential scale and presented in full in Appendix E.

#### Results

##### Initial analyses

As expected, the respondents rated social-orientation higher in the social-orientated condition [M_social_ = 5.73, SD_social_ = 0.81; M_task_ = 4.67, SD_task_ = 1.12; *t*(183) = −7.384, *p* < 0.001, Cohen’s *d* = 1.09], and task-orientation higher in the task-oriented condition [M_social_ = 2.94, SD_social_ = 0.79; M_task_ = 5.15, SD_task_ = 0.82; *t*(183) = 18.64, *p* < 0.001, Cohen’s *d* = 2.74], and the respondents perceived the scenarios as highly realistic across the communication style manipulations [M_social_ = 6.05, SD_social_ = 0.57; M_task_ = 6.03, SD_task_ = 0.65; *t*(183) = 0.235, *p* = 0.814, Cohen’s *d* = 0.03]. In addition, independent *t*-test showed that gender had no relationships with the manipulation check (*p* > 0.1).

##### Correlation analysis

Table 3 shows correlations: customer satisfaction was significantly positive correlated with warmth perception (*r* = 0.67, *p* < 0.01) and competence perception (*r* = 0.15, *p* < 0.05). Warmth perception and competence perception were significantly positively correlated (*r* = 0.18, *p* < 0.05). There are no significant correlations between attachment anxiety and customer satisfaction (*r* = −0.10, *p* > 0.05), warmth perception (*r* = −0.003, *p* > 0.05), and competence perception (*r* = 0.003, *p* > 0.05).

**Table 3:** Descriptive statistics and correlations for Study 2.

Variables	Mean	Standard deviation	Minimum	Maximum	1	2	3	4
1. Warmth perception	5.14	1.03	2.00	7.00	1.00			
2. Competence perception	5.52	0.52	2.50	6.25	0.18^*^	1.00		
3. Customer satisfaction	5.51	0.78	2.33	7.00	0.67^**^	0.15^*^	1.00	
4. Attachment anxiety	3.88	1.28	2.00	6.50	−0.03	0.003	−0.10	1.00

^*^p < 0.05, ^**^p < 0.01.

##### Moderating effect of attachment anxiety

**Figure 3:**
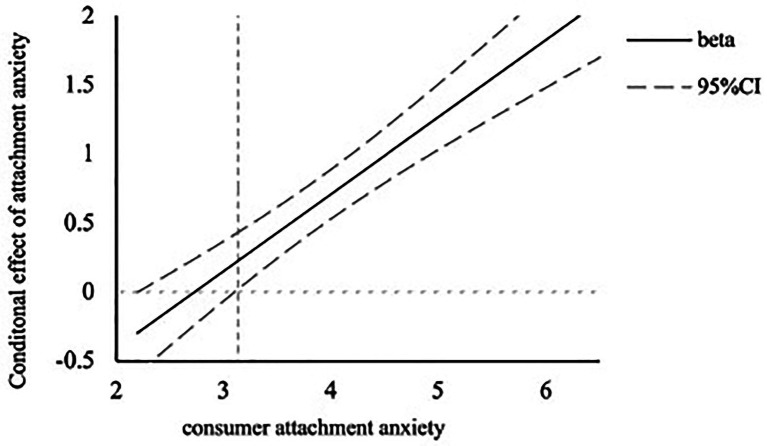
The Johnson- Neyman analysis of consumer attachment anxiety for Study 2.

To verify our hypothesis that attachment anxiety more than attachment avoidance moderates the relationship between a social-oriented communication style and customer satisfaction, we conducted one regression analysis of customer satisfaction as the dependent variable, communication style, mean-centered attachment anxiety scores, mean-centered attachment avoidance, and their interaction with communication style as the independent variables. Results showed a significant positive effect of the communication style on customer satisfaction (*β* = 0.67, *t* = 7.21, *p* < 0.001), with an increase of customer satisfaction when the chatbot used a social-oriented communication style. Importantly, as predicted, the two-way interaction between communication styles and attachment anxiety was significant (*β* = 0.47, *t* = 7.02, *p* < 0.001). We confirmed this result using the Johnson-Neyman analysis for the significant regions, where the cutoff value for the consumer attachment anxiety score equals 3.13 (Figure 3). In other words, subjects scoring high on attachment anxiety felt more satisfied when the chatbot uses a social-oriented communication compared to a task-oriented communication style, while no significant difference was found for participants low on attachment anxiety, suggesting that communication styles did not impact customer satisfaction for this group. Further, consistent with our prediction, there is no significant interaction between communication style and attachment avoidance (*β* = 0.04, *t* = 0.37, *p* = 0.71).

##### Moderated mediation analysis

To test our focal hypothesis that attachment anxiety more than attachment avoidance moderates the relationship between a social-oriented communication style and customer satisfaction because attachment anxiety is more strongly associated with people’s responses to closeness (with warmth perception acting rather than competence perception acting as a mediator), we performed two moderated mediation analyses (PROCESS model 7; Hayes, 2018), incorporating communication styles as the independent variable, warmth perception and competence perception as the parallel mediators, and the two controls as covariates. Mean-centered attachment anxiety and mean-centered attachment avoidance were used as moderators for one of each analysis.

The results showed that attachment anxiety rather than attachment avoidance moderated the indirect effect through warmth perception. Specifically, the index of moderated mediation for attachment anxiety was significant and positive [*β* = 0.27, SE = 0.054, 95% CI (0.17, 0.38)], with the indirect effect being significant for subjects scoring high on attachment anxiety [*β* = 0.82, SE = 0.15, 95% CI (0.54, 1.12)] and non-significant for those scoring low [*β* = 0.07, SE = 0.08, 95% CI (−0.08, 0.24)], whereas the index of moderated mediation for attachment avoidance was non-significant [*β* = 0.031, SE = 0.07, 95% CI (−0.11, 0.17)]. With regard to the indirect effects *via* competence perception, consistent with our hypothesis, the indices of moderated mediation for both attachment anxiety [*β* = –0.003, SE = 0.01, 95% CI (−0.03, 0.01)] and attachment avoidance [*β* = –0.0014, SE = 0.011, 95% CI (−0.022, 0.024)] were not significant.

Study 2 highlights the moderating effect of attachment anxiety and the process driving this effect. First, consistent with Study 1, a social-oriented communication style boosts customer satisfaction. Second, the results further reinforce the importance of attachment anxiety in these effects. As hypothesized, a social-oriented communication style only increases satisfaction through the warmth perception for subjects scoring high on attachment anxiety. In support of our focal hypothesis, this pattern did not emerge for attachment avoidance. Together with the results from Study 1, these findings indicate that attachment anxiety has a unique impact on people’s responses to social communication from the chatbot.

### Study 3

Study 3 is a simulated real-life conversation between a consumer and a chatbot aimed to add support for all our hypotheses (H1, H2, and H3), and further enhance the generalizability of our findings. We controlled for participants’ age and gender.

#### Method

##### Participants

A total of 147 undergraduate students were recruited from a large university in Chengdu, China, to participate in this study in exchange for a nominal payment, of which 58.5% were female, ranging in age from 19 to 24 years (see Appendix D for sample characteristics). No identifiable information was recorded. This sample size has a power greater than 0.95 to detect medium to large effect sizes equivalent to ƒ = 0.3, assuming a conventional alpha level of 0.05 (Cohen, 1988).

##### Study design and procedure

Study 3 was a one-factor (social-oriented vs. task-oriented communication style) between-subject design, with participants’ attachment anxiety and attachment avoidance being measured. We followed the same communication style manipulation and chatbot identity as Study 1 (brand logo, rather than human figure). For this study, researchers design a fictitious website for hotel company called Hamton. Participants were asked to engage with a live chatbot conversation to purchase a room from the fictitious website. Once the live chat initiated, participants were randomly exposed to one of two communication styles, which were manipulated as in our previous studies.

Following the extant procedure (Roy and Naidoo, 2021), the live chat followed the format of Q&A. Consistent with previous studies, the questions were primarily focused on product-related information. The researchers carefully managed the chatbot conversations to ensure that the interactive Q&A between participants and the chatbot focused on the purchasing scenario (Roy and Naidoo, 2021). The live chat on average lasted 5 min, and participants were asked to fill a questionnaire measuring communication style manipulation check, realism checks, customer satisfaction, warmth perception, competence perception, attachment anxiety, and attachment avoidance. Demographic information (age, gender) was also collected. Although the dependent variable was measured, this study can still qualify as a field, as participants engaged with realistic independent variables (e.g., live chat; Roy and Naidoo, 2021).

##### Measurements

We used the same customer satisfaction (*α* = 0.87), warmth perception (*α* = 0.948), competence perception (*α* = 0.85), and communication styles (*α* = 0.869) measures as in Study 1. We used the same items to measure consumer attachment anxiety (*α* = 0.947) and consumer attachment avoidance (*α* = 0.858). All measures used in this study are rated on a seven-point semantic differential scale and presented in full in Appendix E.

##### Pretest

A pretest was conducted to clarify whether a social-oriented communication style triggers the perception of a chatbot as warm or rather task-oriented style hinders it. A neutral (real) condition was included. A total of 60 students from a large university in Chengdu, China, were recruited. They were randomly assigned to one of the three conditions (social-oriented vs. task-oriented vs. neutral) following the same procedure as the main study and they then completed the pretest questionnaire measuring communication style manipulation check and warmth perception.

**Figure 4:**
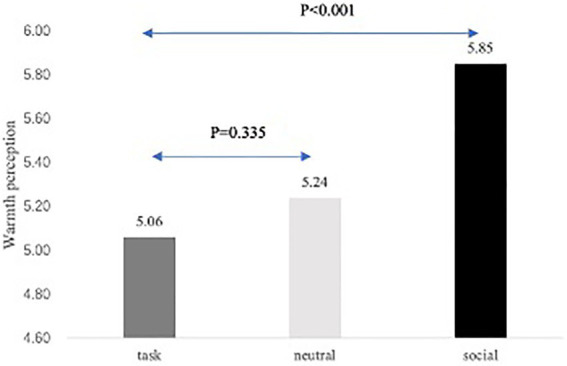
Mean values for warmth perception for pretest.

An ANOVA with the communication style manipulation as the independent variable and communication style manipulation check measure as the dependent variable showed a significant effect of communication manipulation [M_task_ = 5.55, SD_task_ = 0.77; M_neutral_ = 4.13, SD_neutral_ = 0.96; M_social_ = 2.82, SD_social_ = 0.61; *F*(1, 57) = 59.12 *ŋ_p_*^2^ = 0.675, *p* < 0.001]. Importantly, when changing the dependent variable to the warmth perception, the ANOVA showed a significant difference across the three conditions [F(1, 57) = 10.55, *ŋ_p_*^2^ = 0.27, *p* < 0.001; see Figure 4]. Planned contrasts showed that participants in the social condition [M_social_ = 5.85, SD_social_ = 0.49; *t*(57) = 4.375, *p* < 0.001, Cohen’s *d* = −1.13] perceived the chatbot as warmer than did those in the task-oriented condition (M_task_ = 5.06, SD_task_ = 0.64). Task-oriented and neutral conditions did not significantly differ from each other [M_task_ = 5.06, SD_task_ = 0.64; M_neutral_ = 5.24, SD_neutral_ = 0.56; *t*(57) = −0.972, *p* = 0.335, Cohen’s *d* = 0.25], indicating that task-oriented style did not hinder warmth perception.

#### Results

##### Initial analyses

As expected, the subjects rated social-orientation higher in the social-oriented condition [M_social_ = 5.77, SD_social_ = 0.82; M_task_ = 4.57, SD_task_ = 1.11; *t*(145) = −7.443, *p* < 0.001, Cohen’s *d* = 1.23], and task-orientation higher in the task-oriented condition [M_social_ = 2.73, SD_social_ = 0.54; M_task_ = 5.30, SD_task_ = 0.52; *t*(145) = 29.714, *p* < 0.001, Cohen’s *d* = 4.90], and the respondents perceived the scenarios as highly realistic across the communication style manipulations [M_social_ = 6.06, SD_social_ = 0.56; M_task_ = 6.06, SD_task_ = 0.57; *t*(145) = 0.009, *p* = 0.993, Cohen’s *d* = 0.002]. In addition, independent *t*-test showed that gender had no relationships with the manipulation check (*p* > 0.1).

##### Correlation analysis

As Table 4 shows, customer satisfaction was significantly positively correlated with warmth perception (*r* = 0.67, *p* < 0.01). There were no significant correlations between the competence perception and customer satisfaction (*r* = −0.01, *p* > 0.05), competence perception and attachment anxiety, competence perception and warmth perception (*r* = −0.01, *p* > 0.05), attachment anxiety and customer satisfaction (*r* = −0.1, *p* > 0.05), and attachment anxiety and warmth perception (*r* = −0.01, *p* > 0.05).

**Table 4:** Descriptive statistics and correlations for Study 3.

Variables	Mean	Standard deviation	Minimum	Maximum	1	2	3	4
1. Warmth perception	5.14	1.03	2.00	7.00	1.00			
2. Competence perception	5.52	0.52	2.50	6.25	0.11	1.00		
3. Customer satisfaction	5.51	0.78	2.33	7.00	0.67^**^	−0.01	1.00	
4. Attachment anxiety	3.88	1.28	2.00	6.50	−0.01	0.01	−0.09	1.00

^*^p < 0.05, ^**^p < 0.01.

##### Customer satisfaction

**Figure 5:**
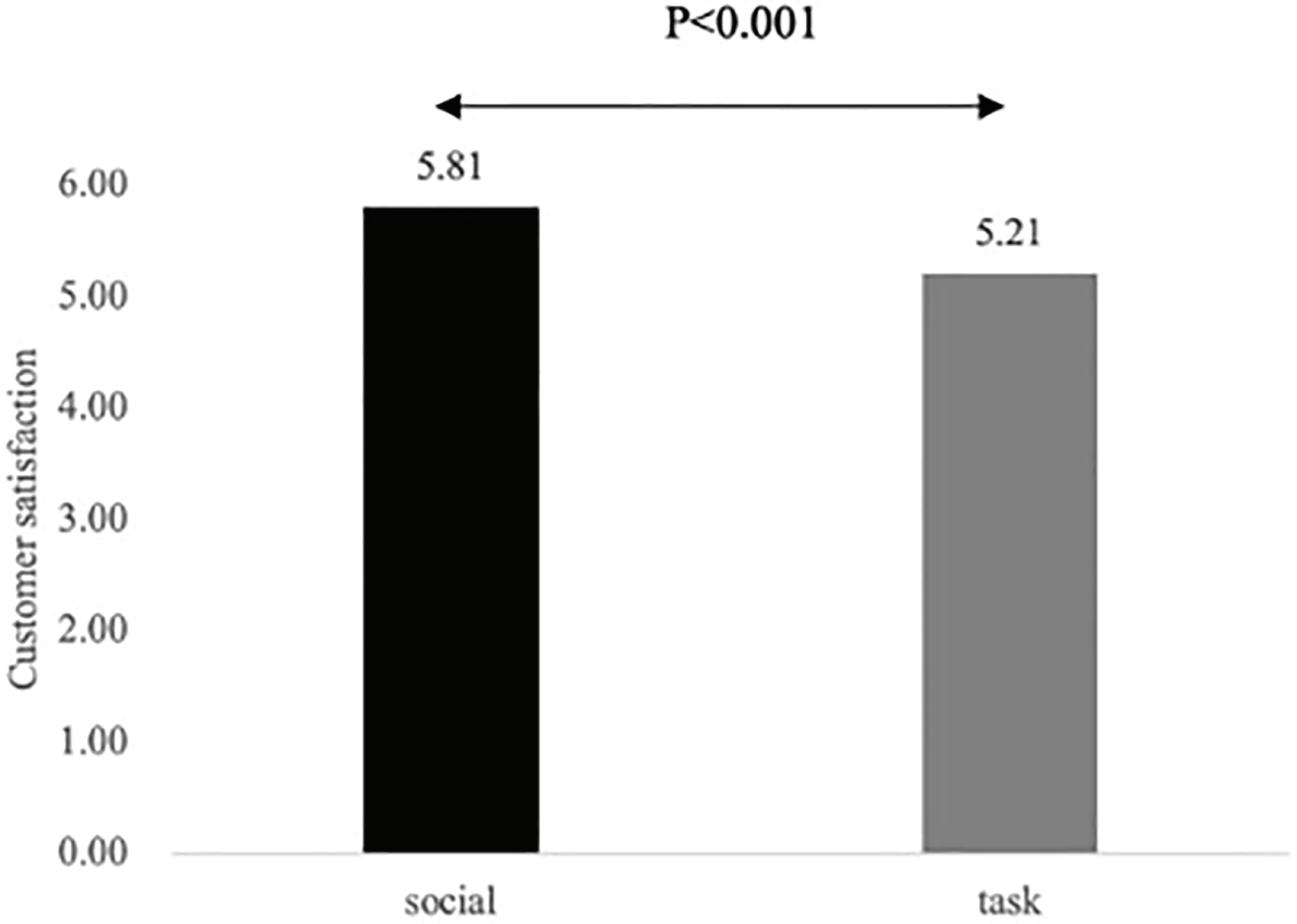
Mean values for customer satisfaction for Study 3.

An ANOVA with customer satisfaction as the dependent variable, the communication style manipulation as the independent variable, and the two controls, revealed a significant main effect of communication style [M_social_ = 5.81, SD_social_ = 0.48; M_task_ = 5.21, SD_task_ = 0.84; *F*(1, 143) = 29.35 *ŋ_p_*^2^ = 0.17, *p* < 0.001], supporting H1 (see Figure 5). The control variables are non-significant (*p* > 0.05).

##### Mediation analysis

To test our mediation hypothesis H2 and rule out competence as a theoretical mediator, we engaged a parallel multiple mediator model (PROCESS model 4; Hayes, 2018) with both warmth and competence perceptions as mediators for the effect of chatbots’ communication style on customer satisfaction. The results confirm that warmth perception mediates the social-oriented communication style’s effect on satisfaction [indirect effect = 0.48, SE = 0.11, 95% CI (0.28, 0.73)]. Social-oriented communication style makes the chatbot appear warmer (*β* = 1.016, SE = 0.14, *t* = 7.21, *p* < 0.001), which boosts satisfaction (*β* = 0.47, SE = 0.055, *t* = 8.64, *p* < 0.001), supporting H2. However, competence perception did not mediate the effect of the social-oriented communication style on satisfaction [indirect effect = 0.004, SE = 0.014, 95% CI (−0.038, 0.019)].

##### Moderating effect of attachment anxiety

**Figure 6:**
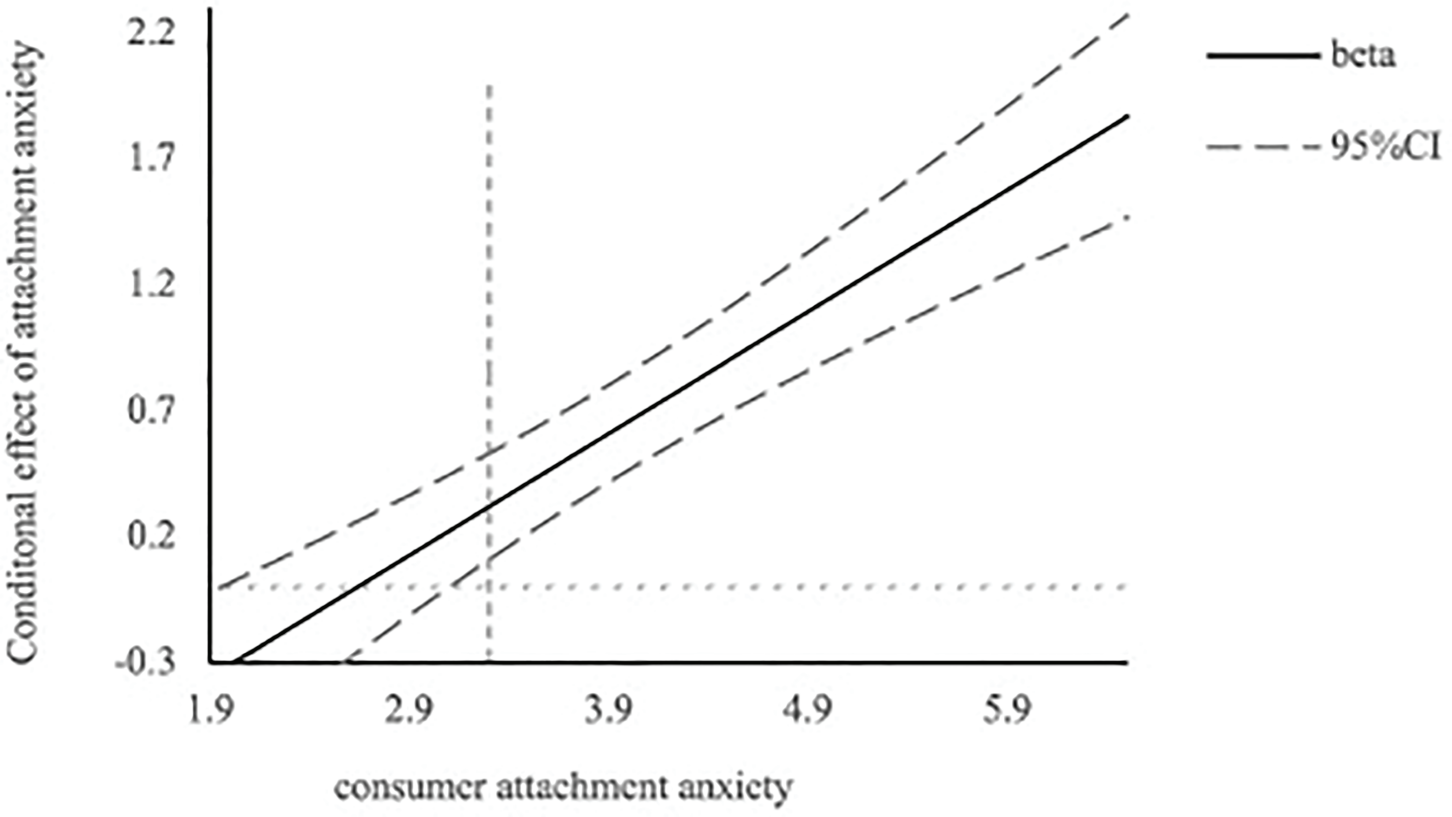
Johnson- Neyman analysis of consumer attachment anxiety for Study 3.

We conducted a regression analysis with the customer satisfaction as the dependent variable, communication style, mean-centered attachment anxiety scores, mean-centered attachment avoidance, and their interaction with communication style as the independent variables. Results revealed a significant main effect of communication style (*β* = 0.65, *t* = 6.57, *p* < 0.001), and interaction of attachment anxiety and communication style (*β* = 0.48, *t* = 6.91, *p* < 0.001). Further, the Johnson-Neyman analysis revealed the cutoff value for the consumer attachment anxiety score equals 3.30 (see Figure 6).

##### Moderated mediation analysis

We performed two moderated mediation analyses (PROCESS model 7; Hayes, 2018), incorporating communication styles as the independent variable, warmth perception and competence perception as the parallel mediators, and the four controls as covariates. Mean-centered attachment anxiety and mean-centered attachment avoidance were used as moderators for one of each analysis.

The results showed that attachment anxiety rather than attachment avoidance moderated the indirect effect through warmth perception. Specifically, the index of moderated mediation for attachment anxiety was significant and positive [*β* = 0.313, SE = 0.068, 95% CI (0.188 0.453)], with the indirect effect being significant for subjects scoring high on attachment anxiety [*β* = 0.993, SE = 0.201, 95% CI (0.618, 1.405)] and non-significant for those scoring low [*β* = –0.067, SE = 0.083, 95% CI (−0.240, 0.089)], whereas the index of moderated mediation for attachment avoidance was non-significant [*β* = 0.043, SE = 0.08, 95% CI (−0.119 0.197)]. With regard to the indirect effects *via* competence perception, consistent with our hypothesis, the indices of moderated mediation for both attachment anxiety [*β* = –0.004, SE = 0.009, 95% CI (−0.023, 0.017)] and attachment avoidance [*β* = –0.002, SE = 0.015, 95% CI (−0.026, 0.036)] were not significant.

Study 3 is an essential aspect of current work. This study was designed following an extant procedure (Roy and Naidoo, 2021) and simulated a real-life discourse between a consumer and the chatbot. This study added further support for our hypotheses which have been tested by the previous two experiments. Based on the work of Roy and Naidoo (2021), we used a naturalistic independent variable that consumers would engage real-life booking hotel situations to replicate the findings from our previous two experiments. In sum, the three studies collectively provide support for internal and external validity.

## Discussion

### Findings

The current research contributes to advancing the understanding of how to ensure positive consumer service experiences with chatbots through the discourse design. Specifically, we show that using a social-oriented (vs. task-oriented) communication style for chatbots promotes customers’ warmth perception of chatbots and ultimately enhances customer satisfaction. However, this process is contingent on consumer attachment anxiety. Supporting our theoretical account, Study 1 provides evidence that a social-oriented communication style boosts customer satisfaction, and the warmth perception of chatbot mediates this effect. Study 2 shows that attachment anxiety moderates these effects. In addition, we chose different products (shoes, jeans, and hotels), varied chatbot identities (no name or Joe), and involved non-student samples. Our findings remained consistent and robust.

### Theoretical implications

The current research provides three important theoretical contributions. First, we contribute to the customer service literature by extending the communication style effect to chatbot service interactions and revealing the psychological process driving the impacts. In contrast to prior research on salesperson effectiveness in handling customer queries, which widely acknowledges that relating and emoting behaviors severely curtail and even neutralize the salesperson’s effectiveness (Marinova et al., 2018; Singh et al., 2018), we show that a chatbot communicating in a social-oriented style improves consumers’ service experiences and boosts their satisfaction.

As such, we also contribute to the hot topic which aims to understand how consumers react to AI that tries to establish and maintain relationships (Huang and Rust, 2021). Our findings show that consumers feel warmer and are more satisfied when these particular chatbots. Thus, this work can serve as a starting point for AI research seeking to engage the consumer by using feeling AI.

Second, we make related contributions to the chatbot humanness literature by demonstrating that chatbots’ social-oriented communication style use can promote the warmth perceptions, which is particularly related to humanness (Gray et al., 2012; Borau et al., 2021). Previous studies show that visual cues, identity cues, gender, and conversational cues (e.g., conversational skill, message interactivity, personality, and conversational style) are useful interventions (e.g., Araujo, 2018; Go and Sundar, 2019; Schuetzler et al., 2020; Borau et al., 2021; Roy and Naidoo, 2021). Recently, the literature began paying attention to the various capabilities that can boost humanness perception, including agency, emotionality, and morality, but few studies actually provide clear guidance on how chatbot should communicate to enhance these perceptions (Söderlund and Oikarinen, 2021). Our findings show that chatbots can enhance perceptions of emotionality (warmth) *via* social-oriented communication. Thus, we also respond to the calls for investigating more design cues to enhance chatbot humanness (Go and Sundar, 2019; Schuetzler et al., 2020; Adam et al., 2021).

Finally, we contribute to the attachment literature by extending the investigation on attachment anxiety effects to chatbot service interactions and demonstrating that attachment patterns developed early in life can guide consumer preferences for chatbots’ specific communication style. Specifically, we show that high anxiety consumers displayed a higher preference for chatbots’ social-oriented communication style and were more satisfied.

### Managerial implications

This study also provides several managerial implications. First, our findings suggest that brand managers can strategically calibrate chatbot communication style to optimize customer experiences and ultimately ensure the sustainability of chatbot technology. For marketing managers, using social-oriented communication for chatbots can make customers feel warmer and enhance their satisfaction. However, this tactic should be approached with caution, as it may not work to certain situations. Thus, it is crucial for brand managers to consider consumer features, namely consumer attachment anxiety. Specifically, social-oriented communication style can enhance customer satisfaction for highly anxiously attached consumers, but this benefit disappears for lowly anxiously attached ones.

Second, our findings provide specific insights into which aspect of the chatbot marketing managers should pay more attention to. Our findings indicate that customers can interpret social cues in chatbot service interaction (i.e., social-oriented communication style) based on the warmth dimension, and are more satisfied with the chatbot. Therefore, managers need to consider how to promote the social, emotional, and relational aspects of chatbots in an effort to positively affect warmth perception.

## Limitations and future research

Our study has some limitations that suggest promising areas for future research. First, we manipulate the chatbot’s communication style as a binary variable, but operationalizing the social-oriented communication style as a continuous variable is also worth exploring. Second, in our three studies, we use a low involvement, low risk, and mainly utilitarian context to test our hypotheses; future research can apply our framework to different contexts to see if the results would replicate. Third, we highlight warmth perception, which distinguishes humans from machines, as the underlying mechanism that drives customer satisfaction. However, unlike prior research that reveals a negative link between challenging human distinctiveness (warmth) and customer attitudes (Gray et al., 2012; Kim et al., 2019), we find that warmth perception is a strong predictor of customer satisfaction. Nevertheless, the belief in human distinctiveness is an interesting and potentially important factor in consumer-chatbot service experiences and should be examined further in future research.

Fourth, other moderators for the documented effects are also worth exploring. Prior work demonstrates that communication mediums and the relationship stages both moderate the interaction between language style and consumer response (Glikson et al., 2017; Moffett et al., 2021). For example, efficiency tends to be less important in the early stages of a customer-brand relationship, but it may prove essential in later stages (Moffett et al., 2021). If this is the case, then customers, who have maintained a long relationship with the brand, may prefer a task-oriented anthropomorphic communication style. Future research should also investigate other individual differences that may moderate the effects of digital virtual assistant communication styles, such as the relationship norm orientation (Li et al., 2019). Specifically, how relationship norm orientation influences individual responses to different anthropomorphic communication styles is worth exploring.

## Data availability statement

The raw data supporting the conclusions of this article will be made available by the authors, without undue reservation.

## Ethics statement

Ethical review and approval was obtained from the Ethics Committee of the Southwestern University of Finance and Economics. All procedures performed in studies were in accordance with the ethical standards of the ethical guidelines of the American Psychological Association. Informed consent was obtained from all the individual participants. We introduced the research purpose, goals, and plans to each participant and ask their permission to participate, and they can withdraw at any time.

## Author contributions

YX: writing—original draft, conceptualization, and visualization. JZ: writing—reviewing and editing. GD: methodology, formal analysis, and investigation. All authors contributed to the article and approved the submitted version.

## Funding

This research was funded by the Doctoral Project of Southwest University of Science and Technology (Grant No. 21sx7109).

## Conflict of interest

The authors declare that the research was conducted in the absence of any commercial or financial relationships that could be construed as a potential conflict of interest.

## Publisher’s note

All claims expressed in this article are solely those of the authors and do not necessarily represent those of their affiliated organizations, or those of the publisher, the editors and the reviewers. Any product that may be evaluated in this article, or claim that may be made by its manufacturer, is not guaranteed or endorsed by the publisher.

## Figures and Tables

**Table:** 

	Task-oriented	Social-oriented
Chatbot anthropomorphic communication style	– Formal conversation – The agent only provides functional information, and focuses on tasks and completing the sale, without providing social conversations, except for the initial greeting and end of session.	– Informal conversation – In addition to offering functional information, the conversation involves relational dialogue with social interactions, such as customary greetings, emotional concerns, social praise, and well-wishing.
Script examples	Chatbot: Welcome to M.com. I am your virtual assistant. What can I help you with? Customer: Are your shoe sizes standard? Chatbot: Based on the feedback from the majority of our customers, the shoe sizes for M sports shoes are standard. I recommend buying the size you usually wear. Chatbot: If you like the shoe, you can place an order and try it on. I believe that M shoes will not disappoint you. Customer: Is the shoe’s material breathable? Chatbot: The net-like shoe surface creates excellent permeability. Chatbot: Please place an order if you like it. If there are more concerns, I recommend buying insurance with the shoe. If it does not fit, the insurance guarantees that you can return the shoes without providing any reason. Customer: Okay, thank you. Chatbot: Thanks for inquiring. Please check out this link for more information about deals and promotions (www.sample.com).	Chatbot: Hi, I’m your friendly and helpful virtual assistant. How good to have you here! I like these sports shoes too! I will be happy to assist you with any questions. Customer: Are your shoe sizes standard? Chatbot: Yes, they are true sizes. You can choose the size you usually wear. Chatbot: Do not worry about the shoe sizes. Customer: Is the shoe’s material breathable? Chatbot: The net-like shoe surface creates excellent permeability. Chatbot: The weather is turning cold, wearing thick socks with the shoe will keep you warm. Please take care of yourself. If you catch a cold, I’ll feel bad. Customer: Okay, thank you. Chatbot: You are so kind. It was so nice to chat with you. Please contact me anytime if have any further questions: www.sample.com. I’m looking forward to our next chat! Hope you have a good day.

**Table:** 

		Frequency	Percentage
Gender	Male	72	36
	Female	128	64
Age	Under 20 years old	84	42
	20–24	116	58

**Table:** 

		Frequency	Percentage
Gender	Male	96	52
	Female	89	48
Age	19–23	74	40
	24–28	72	38.9
	29–33	26	14.1
	Over 33 years old	13	7
Occupation	Student	91	49.2
	State-owned enterprise personnel	18	9.7
	National institution personnel	21	11.4
	Civil servant	5	2.7
	Private enterprise staff	38	20.5
	Foreign company staff	5	2.7
	Other	7	3.8
Education	Associate	5	2.7
	Bachelor	123	66.5
	Master	46	24.9
	Doctor	11	5.9

**Table:** 

		Frequency	Percentage
Gender	Male	86	58.5
	Female	61	41.5
Age	Under 20 years old	54	36.7
	20–24	93	63.3

**Table:** 

Variables	Items	Study	References
Social-oriented	The chatbot is easy to talk with	1, 2, and 3	Williams and Spiro (1985); Van Dolen et al. (2007)
	The chatbot genuinely liked to help me		
	The chatbot seemed interested in me not only as a customer, but also as a human or a person		
	The chatbot liked to talk and put me at ease		
Task-oriented	The chatbot worked hard to provide information	1, 2, and 3	Williams and Spiro (1985); Van Dolen et al. (2007)
	The chatbot primarily focused on the details pertaining to the shoes		
	The chatbot mainly provided she information		
	The chatbot wanted to make sure I made a decision about the shoes		
Warmth perception	The chatbot is warm	1, 2, and 3	Cuddy et al. (2008)
	The chatbot is kind		
	The chatbot is friendly		
	The chatbot is sincere		
Competence perception	The chatbot is competent	1, 2, and 3	Cuddy et al. (2008)
	The chatbot is intelligent		
	The chatbot is capable		
	The chatbot is skillful		
Customer satisfaction	I am satisfied with the chatbot’s provided handling service	1, 2, and 3	Evans et al. (2000)
	I am satisfied with this chatbot as a service assistant		
	Based on my experience, I am satisfied with this service		
Attachment anxiety	I worry that romantic partners will not care about me as much as I care about them	2 and 3	Wang et al. (2017)
	My desire to be very close sometimes scares people away		
	I do not often worry about being abandoned (R)		
	I find that my partner(s) do not want to get as close as I would like		
	I get frustrated if romantic partners are not available when I need them		
	I need a lot of reassurance that I am loved by my partner		
Attachment avoidance	I want to get close to my partner, but I keep pulling back	2 and 3	Wang et al. (2017)
	I am nervous when partners get too close to me		
	I try to avoid getting too close to my partner		
	I usually discuss my problems and concerns with my partner (R)		
	It helps to turn to my romantic partner in times of need (R)		
	I turn to my partner for many things, including comfort and reassurance (R)		

## Appendix A

Chatbot anthropomorphic communication style design.

## Appendix B

Sample characteristics of Study 1.

## Appendix C

Sample characteristics of Study 2.

## Appendix D

Sample characteristics of Study 3.

## Appendix E

Measures used in the two studies.
